# Non-alcoholic fatty liver disease related knowledge among a sample of Egyptians: an exploratory cross-sectional study

**DOI:** 10.3389/fpubh.2023.1290842

**Published:** 2024-05-30

**Authors:** Mona A. Hegazy, Arwa Elshafei, Marwa Rashad Salem, Omar Ashoush, Ahmed Abdelghani

**Affiliations:** ^1^Internal Medicine Department, Faculty of Medicine, Cairo University, Cairo, Egypt; ^2^Public Health and Community Medicine Department, Faculty of Medicine, Cairo University, Cairo, Egypt; ^3^Public Health and Community Medicine Department, Armed Forces College of Medicine, Cairo, Egypt

**Keywords:** awareness, Egypt, general population, NAFLD, questionnaire

## Abstract

**Introduction:**

The global prevalence of Non-alcoholic fatty liver disease (NAFLD) is about 25% worldwide making it an actual health disaster. This study aimed to assess non-alcoholic fatty liver disease (NAFLD)-related knowledge in a sample of Egyptians.

**Materials and methods:**

This exploratory cross-sectional study was conducted on 3,124 individuals using 2000 online and 1,124 printed questionnaire forms. These questionnaires, covering sociodemographic characteristics and fatty liver-related knowledge, comprised 30 items. These items include ten questions on definition, symptoms, and complications: 14 about risk factors, and six about prevention and therapy. The data were analyzed using SPSS. Categorical variables were expressed in proportions and percentages. Chi-square and Fisher’s exact tests were applied as appropriate. For quantitative variables, the t-test, Mann–Whitney U test, Kruskal-Wallis test, and ANOVA test were used for comparisons.

**Results:**

A total of 3,124 respondents were enrolled in the current study. More than half (57%) were females, and 25% ranged in age from 18 to 29. 10.8% of the participants believed that fatty liver patients were asymptomatic, and 34% knew that fatty liver disease was caused by fat accumulation. Regarding predisposing factors, hypercholesterolemia, increased fat in the diet, and obesity had the highest proportion of accurate responses (60, 54, and 46.6%, respectively). On the other hand, 89.3% believed it could be prevented, and 81.4% of the respondents knew that weight reduction could prevent the condition. All respondents (100%) stated wrongly that it was a familial disease related to aging, and most participants (97.3%) did not believe that fatty liver could be treated. Females demonstrated a significantly higher score in preventive measures, while the employed participants scored significantly higher in general knowledge of fatty liver, risk factors, and preventive measures.

**Conclusion:**

Despite the increasing NAFLD prevalence, the current study indicated that Egyptians had fair to moderate knowledge about fatty liver and its risk factors, preventive measures, and therapy. However, a false belief was documented by all respondents that it is a disease that runs in families and occurs only in old age. A fundamental shift in healthcare management with a prioritization of prevention, proactive measures, and early detection of NAFLD should be emphasized.

## Introduction

Non-alcoholic fatty liver disease (NAFLD) is the leading cause of chronic liver disease globally (1). The term “metabolic dysfunction-associated steatosis liver disease” (MASLD) will henceforth refer to NAFLD, describing patients with hepatic steatosis and at least one of five cardiometabolic risk factors considered to have MASLD (2). The prevalence of NAFLD varies in research according to age, gender, ethnicity, and diagnostic methods. Globally, NAFLD prevalence averages 24.1%, ranging from 13.5% in Africa to 31.8% in the Middle East (3). Despite limited data on the extent of NAFLD in Egypt, the available data indicate a prevalence of nearly 32% among Egyptians, compared to a global prevalence of 25% (4).

Egypt ranks among the top ten countries with the highest obesity rates in the world, with about 71.2% of adult men being overweight, 26.4% being obese, 79.4% of adult women being overweight, and 48.4% being obese (5). The hepatic form of the metabolic syndrome, NAFLD, has been associated with chronic kidney disease, obstructive sleep apnea (OSA), type 2 diabetes, and cardiovascular disorders (6). Those who fit the histopathologic criteria for non-alcoholic steatohepatitis, or NASH (hepatic inflammation accompanying hepatic steatosis), are at the highest risk of liver-related cardiovascular morbidity and mortality (7).

Although NAFLD is common, little research has examined public knowledge of the condition. A study conducted by Zhang et al. (8) among office employees in Beijing revealed low awareness of NAFLD among office workers, highlighting the need for more educational efforts to decrease the burden of NAFLD in China. Another study conducted among a sample of the adult Saudi population showed that only 7% had good knowledge of fatty liver. The highly educated participants formed a significantly higher percentage of those with good knowledge (7). A more recent study conducted by Morrill et al. (9) among a community-based sample of Mexican-origin women revealed that low NAFLD awareness and knowledge levels warrant more significant efforts to educate the general population. Similar results were revealed by a study conducted among a sample of adults in Beijing who had low knowledge about NAFLD (10).

Considering that maintaining a healthy lifestyle and losing weight are still the cornerstones of NAFLD care, it is alarming that there is a paucity of knowledge about NAFLD as a preventable illness that may be controlled in its early stages (11). Most studies have focused on the genetic pathways, clinical traits, and test results that cause liver steatosis and fibrosis (9, 12). To our knowledge, this study is the first to assess Egyptians’ comprehension of non-alcoholic fatty liver disease and its risk factors. It could help create instructional materials that promote the prevention, early detection, and treatment of this frequent liver disease. Since the burden of NAFLD will rise in the upcoming decades, addressing contributing factors is essential. Awareness is crucial for this disease because it is reversible and treatable in its early stages, but only if people know they have it and can stop its progression.

## Materials and methods

### Study design and setting

The current exploratory cross-sectional study was conducted among a sample of the general population in Egypt to assess their knowledge and attitudes about fatty liver from September 2021 until March 2022. The research followed the Checklist for Reporting Results of Internet E-Surveys (13) and adhered to the Strengthening the Reporting of Observational Studies in Epidemiology (STROBE) Statement (14).

### Sample size and sample design

Population recruitment was carried out by the researchers using a consecutive convenience sampling strategy. The recruitment process started in September 2021, and the inclusion criteria were that the general population was at least 18 years old and willing to participate in the study. Healthcare providers were excluded; this information was mentioned on the electronic survey cover page before the participants completed the survey. The total number of collected questionnaires was 1,124 printed copies and 2055 online forms obtained via a Google Form link sent through social media. With a high acceptance rate of 97.3%, 2000 final online forms were collected, resulting in 3124 questionnaires.

### Data collection tool

A pre-tested, anonymous questionnaire was used to collect data from the study participants. It included four sections: (a) sociodemographic characteristics, covering age, sex, education, occupation, marital status, and residence; (b) knowledge of fatty liver, assessed through a total of 31 items, including questions on definition, symptoms, and complications (10 questions), risk factors (14 questions), and prevention and therapy (6 questions). The questions were closed-ended, offering yes, no, and do not know options. Accurate knowledge answers were scored 1, while wrong answers or answers with “I do not know” received a score of 0. The total raw score, if all answers were correct, was 31; (c) three attitude questions toward NAFLD; (d) sources of knowledge about fatty liver, including scientific websites, literature, colleagues or health care providers, television, the internet, Facebook, WhatsApp, World Health Organization (WHO) website, Center for Disease Control and Prevention (CDC), Egyptian Ministry of Health and Population (MOHP), and others.

The questions used in these sections were adopted from the available literature (15–17). Two language experts translated the questions into Arabic, and then two independent language experts translated them back into English.

A Google form was created, and participants were invited to complete and submit it. A questionnaire link was shared with groups on Facebook and WhatsApp, especially after the authorities implemented legal processes to achieve social distancing due to COVID-19’s critical situation. However, relying solely on online forms was inappropriate, given that Egypt’s illiteracy rate was 25.8% in 2017, according to the Central Agency for Public Mobilization and Statistics (18). Additionally, online forms would target only the educated and those familiar with using an online questionnaire. Therefore, the researchers printed and distributed the questionnaire, conducting direct interviews using a structured printed questionnaire.

The researchers selected online groups with many members to achieve a high response rate. Initially, requests were sent to the administrators of these groups to get permission for survey dissemination. Subsequently, the researchers posted a link to the survey with a statement including its purpose and encouraging members to participate.

To reduce inter- and intra-observer bias, the data collection team, consisting of two researchers, got a two-hour training session, followed by a post-test to assess their preparation and reporting quality.

#### Pilot test

The data collection tool was tested on 30 employees at the Faculty of Medicine, Cairo University, Internal Medicine Hospital (via a face-to-face interview) and 30 online participants beyond the study sample. This process aimed to test the questionnaire’s language, content, and length suitability. Any variations between online and direct responses, such as impediments and challenges, were investigated, and necessary modifications were applied. Face and content validity were examined by collecting viewpoints from public health experts.

A Cronbach’s alpha reliability test was performed for different sections of the questionnaire and the whole questionnaire. The reliability was 0.84 for the knowledge section, 0.77 for the attitude section, and 0.72 for the whole questionnaire.

#### Statistical analysis

The researchers conducted a quality check of the gathered data in the field (for the printed forms), followed by data entry using SPSS version 24.0 (IBM, SPSS, United States). Categorical variables were expressed as proportions and percentages. Chi-square and Fisher’s exact tests were applied as appropriate. For quantitative variables, normality was examined, and results were expressed using the mean, median, and standard deviation. Comparisons were performed using the t-test, Mann–Whitney U, Kruskal-Wallis, and ANOVA tests. A *p*-value <0.05 was considered significant; all significance tests were two-tailed.

#### Ethical considerations

The Faculty of Medicine at Cairo University’s ethical review board revised and approved the study protocol (N-71-2021) in August 2021. Inclusion in the study was limited to those who agreed to participate. The Helsinki Declaration of Biomedical Ethics guides all data collection methods (19). All study participants provided written informed consent. For the online form, participants provided electronically signed informed consent. Those who declined to participate by submitting an empty form after answering “Not willing to participate” were excluded. Data confidentiality was maintained throughout the study.

## Results

A total of 3,124 respondents were enrolled in the current study. More than half (57%) were females, and 25% ranged in age from 18 to 29. Rural residents represented less than 3% of the studied group, while those with a higher education comprised 43%. About one-third were diabetic (32.5%).

Ten questions assessed the general knowledge about fatty liver. Only one-third of participants know that fat deposits in the liver cause fatty liver. Moreover, fatty liver is a prevalent disease in Egypt, with the majority (84.3%) having an incorrect answer, suggesting that fatty liver could be due to HCV or HBV. Only 18.6 knew that fatty livers can cause liver cell cancer. About three-quarters (72.2%) correctly knew that patients with fatty livers complained of general fatigue. The majority did not know that fatty liver patients are asymptomatic. The minimum and maximum scores for general knowledge were 0 and 10, respectively, with a median of 1 and an interquartile range of 3. Thus, less than one-third of the studied group answered the questions correctly, as illustrated in Table 1.

**Table 1:** Fatty liver-related general knowledge among the enrolled participants (*N* = 3,124).

General knowledge items	*n* (%)
*1. Fat deposits in the liver are the cause of fatty liver*	
Yes	1,068 (34.2)
No	2055 (65.8)
*2. Fatty liver is a prevalent disease in Egypt*	
Yes	908(29.1)
No	2,216(70.9)
*3. Fatty liver could be due to HCV or HBV*	
Yes	492 (15.7)
No	2,632 (84.3)
*4. Fatty liver causes liver cirrhosis*	
Yes	1,442(46.2)
No	1,682(53.8)
*5. Fatty liver can lead to significant health problems*	
Yes	1710(54.7)
No	1,414(45.3)
*6. Fatty liver can cause live cell cancer*	
Yes	582(18.6)
No	2,542(81.4)
*7. Fatty liver is related to cardiac problems*	
Yes	1,002(32.1)
No	2,122(67.9)
*8. Patient with fatty liver complains of general fatigue*	
Yes	867(27.8)
No	2,257(72.2)
*9. Patient with fatty liver has right hypochondriac pain*	
Yes	456(14.6)
No	2,668(85.4)
*10. Patient with fatty liver is asymptomatic*	
Yes	338(10.8)
No	2,786(89.2)
*General Knowledge Score Median (IQR)*	1 (3)

Participants’ knowledge about the predisposing risk factors for fatty liver is presented in Table 2. It was assessed through fourteen questions. Surprisingly, despite 46.6, 54.1, and 60.1% correctly stating that fatty liver is related to obesity, high fat intake in the diet, and high blood levels of cholesterol, respectively, all the participants agreed that familial predisposition and aging are non-modifiable risk factors, which is a wrong assumption. The maximum score was 12, and the minimum was 0, with a median of 4 and an interquartile range (IQR) of 6.

**Table 2:** Risk factors for developing fatty liver-related knowledge among the enrolled participants (*N* = 3,124).

Knowledge about risk factors for developing fatty liver	*n* (%)
*1. Aging is a risk factor for fatty liver*	
Yes	3,124 (100.0)
No	0 (0.0)
*2 Fatty liver runs in families*	
Yes	3,124 (100.0)
No	0 (0.0)
*3. Fatty liver is related to obesity*	
Yes	1,455 (46.6)
No	1,669(53.4)
*4. Fatty liver is related to diabetes*	
Yes	967 (31.0)
No	2,157 (69.0)
*5. Fatty liver is related to hypercholesterolemia*	
Yes	1878 (60.1)
No	1,246 (39.9)
*6. Fatty liver is related to hypertension*	
Yes	1,381 (44.2)
No	1743 (55.8)
*7. Fatty liver is related to overeating*	
Yes	1,269 (40.6)
No	1855 (59.4)
*8. Fatty liver is related to physical inactivity*	
Yes	1,291 (41.3)
No	1833 (58.7)
*9. Fatty liver is related to increased fat intake*	
Yes	1,690 (54.1)
No	1,434 (45.9)
*10. Fatty liver is related to increased oil intake*	
Yes	1,472 (47.1)
No	1,652 (52.9)
*11. Fatty liver is related to increased sugar intake*	
Yes	1,381 (44.2)
No	1743 (55.8)
*12. Fatty liver is related to antibiotic over-usage*	
Yes	530 (17.0)
No	2,594 (83.0)
*13. Fatty liver is related to few sleeping hours*	
Yes	525 (16.8)
No	2,599 (83.2)
*14. Fatty liver is related to alcohol intake*	
Yes	877 (28.1)
No	2,247 (71.9)
*Risk Factors Knowledge Median (IQR)*	4 (6)

Most of our participants (89.3%) expressed perfect knowledge that fatty liver could be prevented, 84.5% through a healthy diet, 81.4% through weight loss, and 51.2% through regular physical activity. Moreover, 97.3% of participants, meaning over 3,000 Egyptians from different governmental areas, believed there was no treatment for fatty liver once it occurred. Table 3 illustrates that the mean treatment and prevention knowledge score was 4.0 ± 1.2, with a maximum score of 6 and a minimum of 0.

**Table 3:** Fatty liver preventive measures and treatment line-related knowledge among the enrolled participants.

Prevention and management-related knowledge	*n* (%)
*1. Fatty liver could be prevented*	
Yes	2,789 (89.3)
No	335 (10.7)
*2. Fatty liver could be prevented by weight reduction*	
Yes	2,542 (81.4)
No	582 (18.6)
*3. Fatty liver could be prevented by regular physical activity*	
Yes	1,598 (51.2)
No	1,526 (48.8)
*4. Fatty liver could be prevented by a healthy diet*	
Yes	2,640 (84.5)
No	484 (15.5)
*5. There is a treatment for fatty liver*	
Yes	83 (2.7)
No	3,041 (97.3)
*6. Fatty liver could be easily managed if early diagnosed*	
Yes	2,863 (91.6)
No	261 (8.4)
*Prevention Score Median (IQR)*	4 (1)

The fatty liver-related mean scores were compared between males and females, as shown in Table 4. Females demonstrated significantly higher scores in preventive measures (*p* = 0.002). Table 5 reveals that the working participants revealed significantly higher scores regarding general knowledge, risk factors, and preventative measures, with *p* < 0.001, 0.001, and 0.003, respectively.

**Table 4:** Fatty liver-related mean knowledge scores among males and females.

Item	Male (*N* = 1,325)	Female (*N* = 1796)	*p*-value
*Knowledge (mean ± SD) “Score 10”	2.4 ± 2.5	3.1 ± 2.7	**0.9**
*Risk (mean ± SD) “Score 14”	4.1 ± 3.6	5.1 ± 3.8	0.6
**Prevention (mean ± SD) “Score 7”	4.0 ± 1.19	4.1 ± 1.23	**0.002**

*Mann–Whitney test; **Independent samples *t*-test. Bold values indicate significant.

**Table 5:** Fatty liver-related mean knowledge scores according to working status.

Items	*Knowledge score “10”	*Risk score “14”	**Prevention score “7”
Not working (*N* = 1,056)	2.4 ± 2.7	4.3 ± 3.8	3.9 ± 1.2
Working (*N* = 1855)	3.1 ± 2.6	5.04 ± 3.6	4.06 ± 1.1
Retired (*N* = 213)	2.3 ± 2.6	4.1 ± 3.7	3.8 ± 1.4
*p*-value	<0.001	<0.001	0.003

*Kruskal – Wallis test; **ANOVA test.

Fatty liver’s overall mean knowledge score was 10.03 ± 4.99, with a median of 10 and an IQR of 8, while the highest score reached was only 58%. Furthermore, the results obtained from males and females were comparable (*p* = 0.8). However, working participants scored (10.9 ± 4.8) significantly higher than non-working (8.7 ± 4.9) and retired (8.6 ± 5.2), with *p* < 0.001.

**Figure 1:**
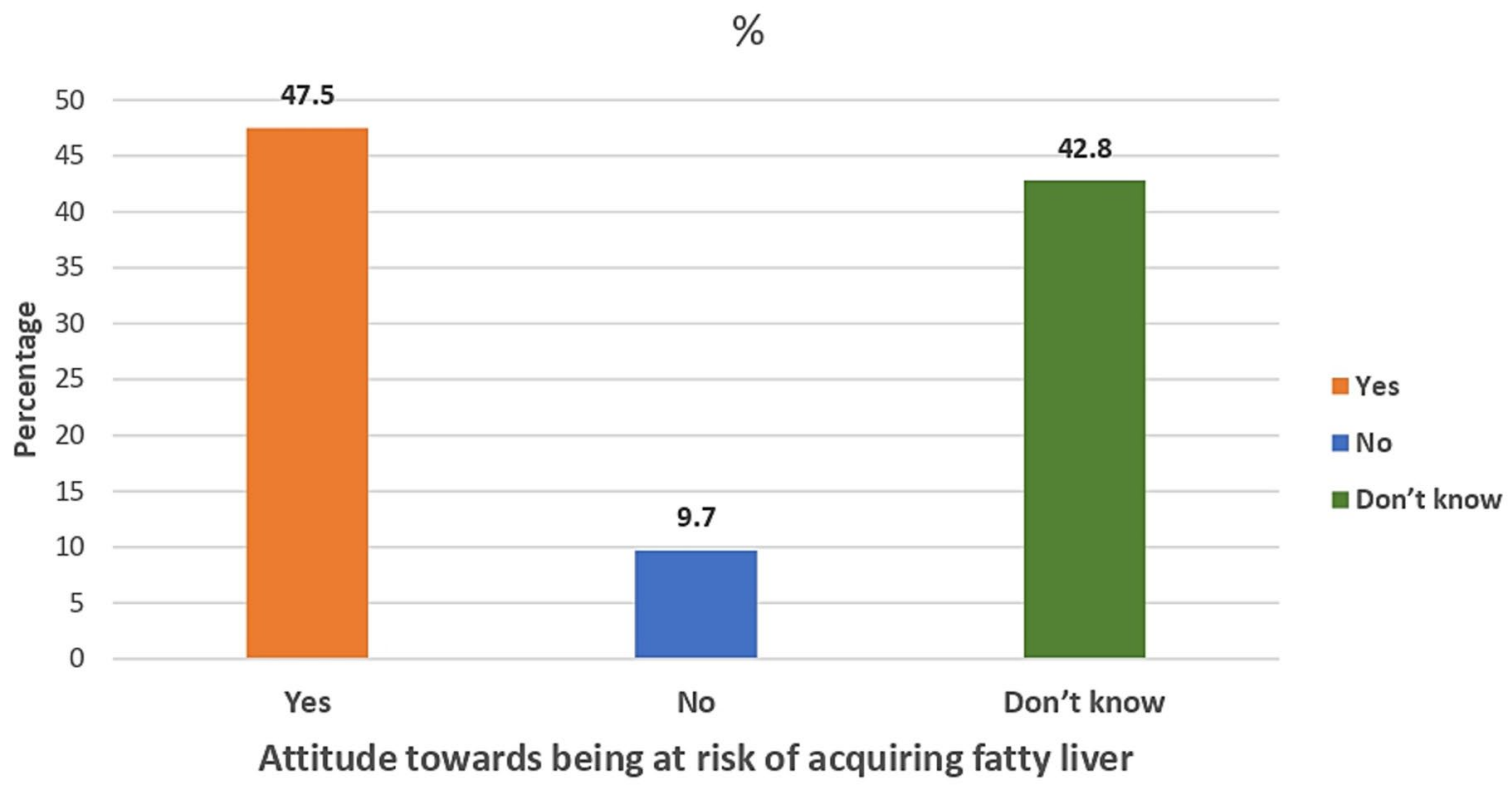
Percent distribution of the enrolled participants by their attitude towards being at risk of acquiring fatty liver (*N* = 3,124).

**Figure 2:**
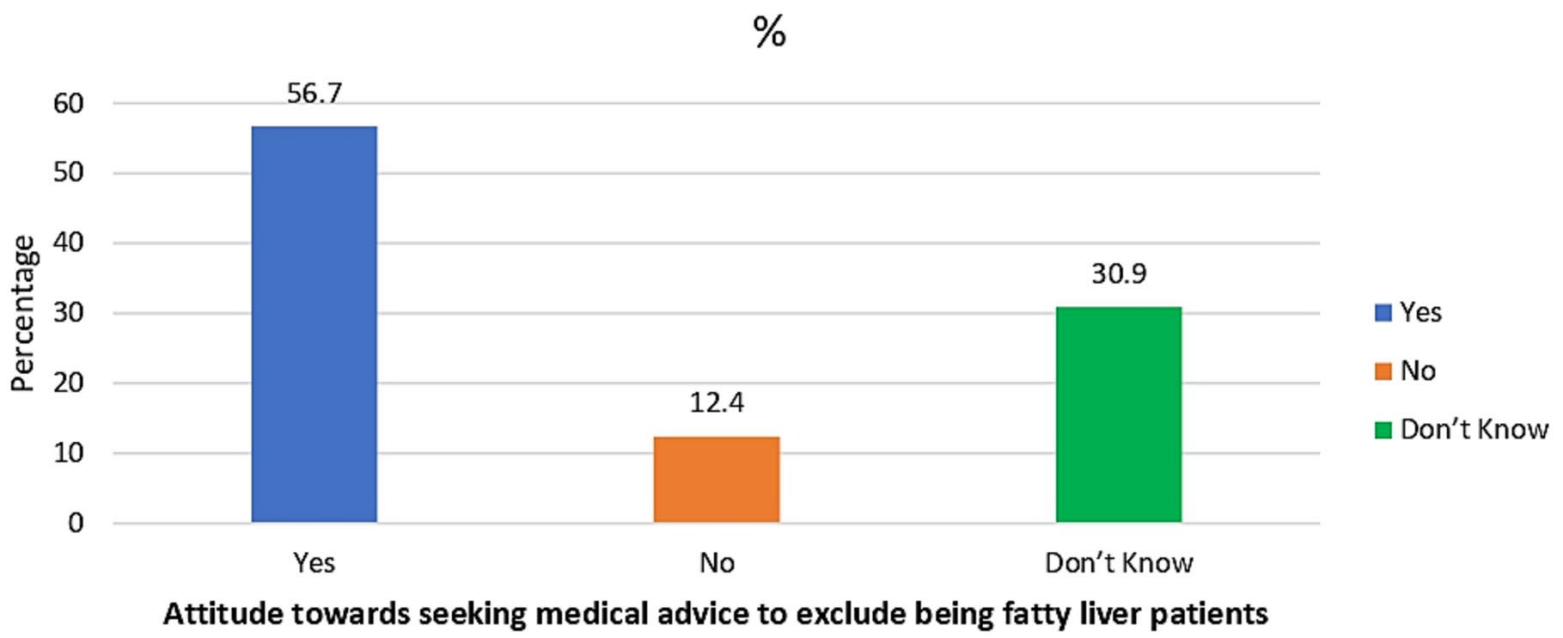
Percent distribution of the enrolled participants by their attitude towards seeking medical advice to exclude being fatty liver patients (*N* = 3,124).

**Figure 3:**
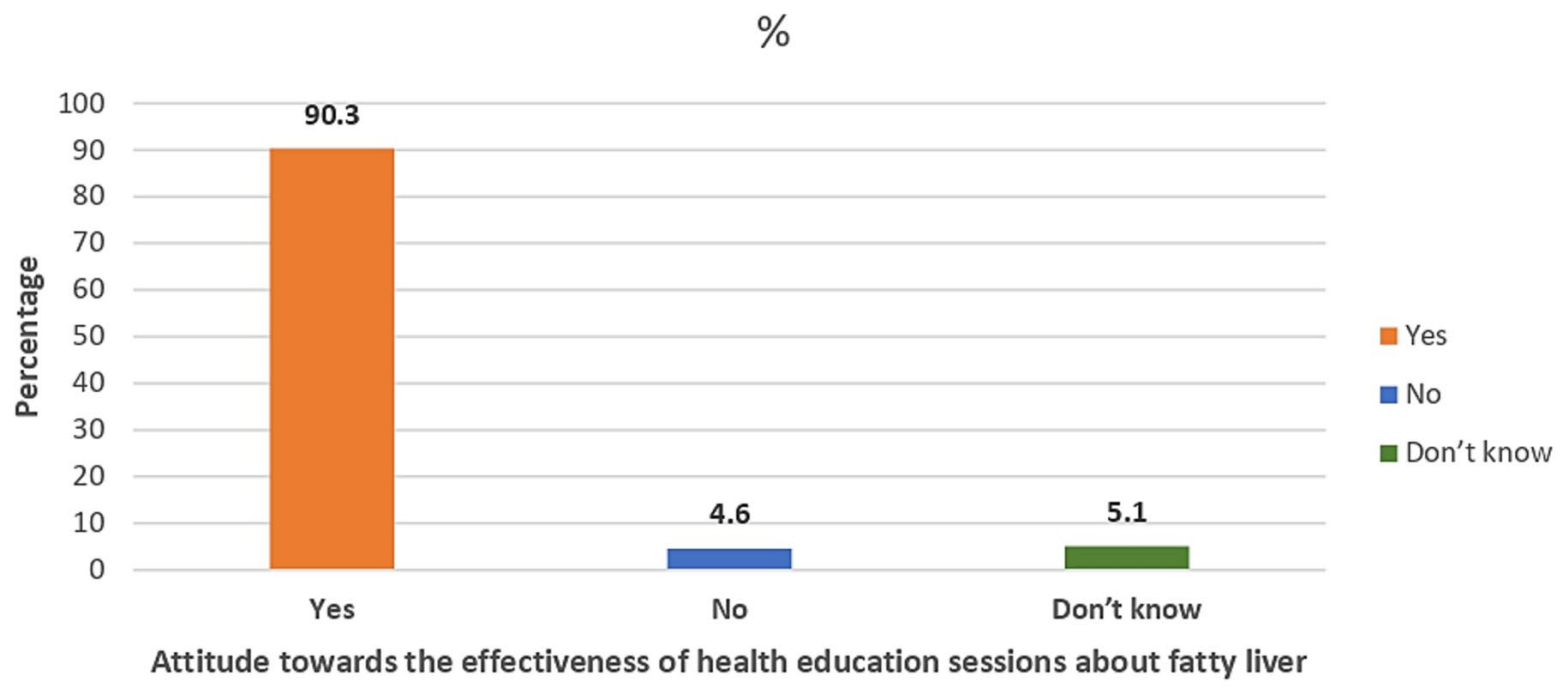
Percent distribution of the enrolled participants by their attitude towards the effectiveness of health education sessions about fatty liver (*N* = 3,124).

**Figure 4:**
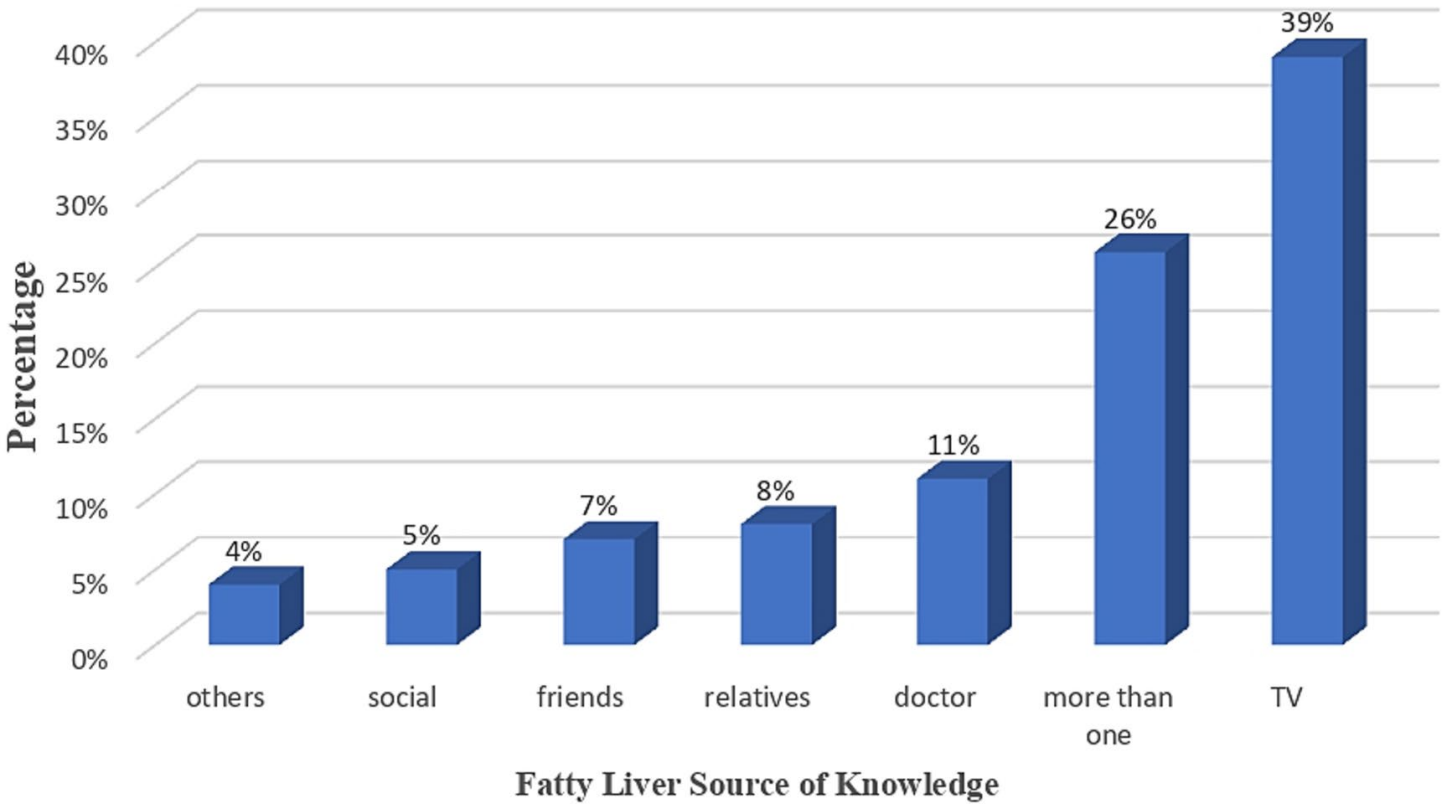
Source of Fatty liver knowledge among the enrolled participants.

Figure 1 illustrates that less than half (47.5%) of the enrolled participants considered themselves at risk of acquiring fatty liver. However, more than half (56.7%) of the participants were willing to seek medical advice to ensure they were not fatty liver patients, as shown in Figure 2. Additionally, Figure 3 reveals that over 90% acknowledged the importance of health education sessions about NAFLD. The primary source of knowledge is television, as reported by more than one-third of the enrolled participants (39%). On the other hand, only 11% reported physicians as a source of their NAFLD-related knowledge (Figure 4).

## Discussion

In the present study, it was interesting that most Egyptian participants agreed that NAFLD health education workshops are essential and that NAFLD could be prevented. Weight loss was acknowledged as an effective preventive measure by 81% of participants. More than half of the study participants reported that fatty liver is related to hypercholesterolemia and increased fat in the diet. Seeking medical assistance to make sure they were not patients with fatty liver was a perfect response from 56.7% of our participants based on their response of considering themselves at risk of having fatty liver. However, unfortunately, all the participants agreed that familial predisposition and aging are the main risk factors.

Despite a knowledge base of 54.7% that fatty liver can lead to serious health problems, only 34.2% of the study participants were aware that it is a common condition in Egypt. Compared to our study, a higher percentage (71.2%) of subjects in the Singapore study had heard of NAFLD. However, this difference is likely related to the Singapore study in a health forum focused on lifestyle and digestive diseases (17). Furthermore, our findings show that no regional or national NAFLD policies are in place.

Regarding risk factors, 53.4% of the current study participants did not think obesity was a risk factor for NAFLD. Furthermore, more than 58.7% of respondents did not believe that a lack of physical exercise was a cause of NAFLD. This finding highlights the need for better information about the likelihood of developing these liver issues and the specific risk factors for this worldwide health issue.

A previously published review article indicates that non-alcoholic fatty liver disease and type 2 diabetes often coexist, with NAFLD prevalence in 59.67% of T2DM patients (20). Despite the apparent correlation between NAFLD and type 2 diabetes, 69% of participants in the current study did not perceive diabetes as a risk factor for fatty liver. Our findings align with those reported by Alsubaie et al. (21); only 17.2% believe that diabetes and NAFLD are associated.

NAFLD has swiftly emerged as the predominant chronic liver disease in children and adolescents (21). Unfortunately, our participants did not consider that children may have fatty livers and that aging increases the risk for NAFLD progression. According to a study by Nobili and colleagues, people with NAFLD tend to be older, have elevated body mass indices, increased waist circumferences, and more metabolic risk factors (22). However, it is a disease of all age groups who possess the risk factors.

In our survey, 2,786 participants (89.2%) believed that the fatty liver must be accompanied by symptoms and selected different symptoms as patient presentations, such as right hypochondriac discomfort (14.6%) and general fatigue (27.8%). This was the wrong population concept, as hepatic steatosis is often accidentally found and identified through liver ultrasonography or elevated liver biochemical tests linked to non-alcoholic fatty liver disease. The majority of NAFLD patients show no symptoms, while some might experience malaise, sluggishness, or slight pain in the right upper quadrant (23).

About half of the enrolled participants agreed that increased oil, fat, and sugar consumption increased the risk of fatty liver disease. The rising consumption of beverages with added sugar and high-fructose corn syrup has been linked to the rising prevalence of NAFLD, notably non-alcoholic steatohepatitis (NASH). It is becoming more evident that the intestinal microbiota contributes to the development of NAFLD as we learn more about the close connection between the gastrointestinal tract and the liver. Interestingly, fructose consumption and obesity have been connected to endotoxins generated by the human gut (24). Our team has previously evaluated the effects of lifestyle and food choices on gut flora, significantly affecting the immune system. According to the ESSAP and nutrition and lifestyle gut microbiota modifier health scores, one can maintain a favorable gut microbiome environment by participating in at least 20 min of daily exercise, getting at least 8 h of sleep every night, consuming less than two teaspoons of sugar daily, eating foods high in prebiotics, and avoiding antibiotic use (25).

Notably, the majority (89.3%) believed NAFLD could be prevented, a finding consistent with a study conducted among a sample of the Saudi population, in contrast to another study where the rate was only 2% (21). Most survey participants agreed that losing weight and maintaining a healthy diet might prevent fatty liver disease. On the other hand, only 83 people (2.7%) thought it was a treatable condition. The best management strategy for NAFLD has not yet been established, despite the widespread agreement that individuals with NASH should get treatment, especially when it comes to fibrosis. The mainstay of treatment for NAFLD is lifestyle adjustment, including dietary changes, continued weight loss, and increased activity (26). A meta-analysis found that while non-alcoholic steatosis (NAS) is improved by weight loss of greater than 7%, hepatic steatosis is only improved by weight loss of 5% (27). Nutritional counseling and weight loss through calorie restriction have been shown to improve liver histology through several randomized controlled trials. Potential lifestyle changes include cutting calories to reduce weight, altering the diet by consuming different macronutrients, and increasing physical activity, such as resistance and aerobic exercise (12).

In the current study, females demonstrated significantly higher scores in knowledge items related to preventive measures. This contrasts with a recent study conducted among a sample of the Saudi population, where there was no significant relationship between knowledge level and participants’ gender (26). An earlier study conducted in the US also found no significant difference between males and females regarding their awareness of NAFLD ().

The working participants in our study had considerably higher scores for NAFLD than the non-working or retired people regarding general knowledge, risk factors, and preventative measures. This is consistent with the findings of Alhumaid. and colleagues, who found that working people with a bachelor’s degree demonstrate much better awareness levels (21).

## Conclusion

NAFLD knowledge was uneven across the survey domain among the different groups sampled. Significant differences were observed based on awareness levels regarding gender, education level, and occupation. Surprisingly, none of the participants knew that children might have NAFLD.

A fundamental shift must occur in which health promotion, prevention, proactive measurements, and early detection of NAFLD replace the current emphasis on managing end-stage liver disease complications.

### Limitations

The current study findings should be viewed in light of the following limitations: using non-probability sampling techniques. However, the researchers conducted the current study to explore this new area of inquiry and generate hypotheses, as there is limited information available about NAFLD knowledge among the general population. Nevertheless, the study provides more insights into a poorly researched area and contributes to guiding future nutrition education programs. We do recommend conducting further studies on a national basis.

Second, bias in recall inevitably exists in a cross-sectional observational study. However, we have taken measures such as piloting the questionnaire and shortening the recall period to minimize its impact.

Third, sampling bias was a concern; however, we attempted to minimize the bias by using both printed and online forms to reach different population strata.

### Recommendation

Non-alcohol fatty liver disease requires collaboration among hepatologists, diabetologists, cardiologists, nutritionists, and general practitioners (GPs) and a public health effort focused on prevention. Educational strategies and campaigns, including media initiatives, should be used to promote NAFLD awareness, especially among high-risk persons, for prevention, early detection, and treatment. Comprehensive guidance from physicians can be of paramount importance in NAFLD prevention.

## Data availability statement

The raw data supporting the conclusions of this article will be made available by the authors, without undue reservation.

## Ethics statement

The studies involving humans were approved by the ethical review board at the Faculty of Medicine, Cairo University, revised and approved the study protocol (N-71-2021 on August 2021). Only those who agreed to participate were included in the study. The Helsinki Declaration of Biomedical Ethics was applied to all data collection methods. All the study participants provided written informed consent. For the online form, the study participants provided electronically signed informed consent. Those who declined to participate by submitting an empty form after answering "Not willing to participate" were excluded. Data confidentiality was maintained throughout the study. The studies were conducted in accordance with the local legislation and institutional requirements. The participants provided their written informed consent to participate in this study.

## Author contributions

MAH: Conceptualization, Data curation, Project administration, Resources, Supervision, Validation, Visualization, Writing – original draft, Writing – review & editing. AE: Formal analysis, Methodology, Software, Writing – review & editing. MRS: Data curation, Methodology, Formal Analysis, Validation, Writing – original draft, Writing – review & editing. OA: Data curation, Resources, Validation, Writing – review & editing. AA: Project administration, Validation, Visualization, Software, Writing – original draft.
